# Microwave-Assisted Freeze-Drying of Monoclonal Antibodies: Product Quality Aspects and Storage Stability

**DOI:** 10.3390/pharmaceutics11120674

**Published:** 2019-12-12

**Authors:** Julian Hendryk Gitter, Raimund Geidobler, Ingo Presser, Gerhard Winter

**Affiliations:** 1 Department of Pharmacy, Pharmaceutical Technology and Biopharmaceutics, Ludwig-Maximilians-Universität München, 81377 Munich, Germany; 2 Boehringer Ingelheim Pharma GmbH & Co. KG, Pharmaceutical Development Biologicals, 88397 Biberach an der Riß, Germany

**Keywords:** freeze-drying, lyophilization, drying, microwave, protein, monoclonal antibody, stability

## Abstract

In order to overcome the downside of long conventional freeze-drying (CFD) process times for monoclonal antibody formulations, microwave-assisted freeze-drying (MFD) was introduced. Recently, the general applicability and potential shortening of drying times were shown. However, little is known about the storage stability of MFD products compared to CFD references. Additionally, batch homogeneity issues were seen within MFD in the past. In this study, we examined four different formulations of two different monoclonal antibodies using three different glass-forming excipients: sucrose, trehalose, and arginine phosphate. These formulations were freeze-dried with two different drying protocols (CFD and MFD), stored for 24 weeks, and analyzed for solid-state and protein-related quality attributes. Moreover, a new microwave generator setup was investigated for its potential to improve batch homogeneity. In all investigated formulations, comparable stability profiles were found, although the classical magnetron generator led to inferior batch homogeneity with respect to residual moisture distribution. In contrast, the new MFD setup indicated the potential to approximate batch homogeneity to the level of CFD. However, for future applications, there is an unabated need for new machine designs to comply with pharmaceutical manufacturing requirements.

## 1. Introduction

Conventional freeze-drying (CFD), also referred to as lyophilization, is a gentle drying method to improve the long-term stability of pharmaceuticals, specifically of protein drugs [1]. The method has been used for pharmaceutical industrial purposes since World War II, for the preparation of human blood plasma [2], and the demand for freeze-drying (FD) remains high. By 2018, one-third of all parenteral protein formulations approved by the European Medicines Agency were freeze-dried products [3]. During lyophilization, the protein drug is immobilized in the solid-state, slowing down chemical and physical degradation reactions [2,4,5,6,7]. Additionally, freeze-dried solids may have other benefits with respect to shipping and storage [8]. 

In general, freeze-drying comprises three steps: freezing, primary drying (= sublimation drying), and secondary drying (= desorption drying). Typically, the sublimation step is widely described to be the most time-consuming, and conventional freeze-drying is associated with lengthy process times [2,9,10,11,12]. One alternative drying method utilizing microwaves is known from the food industry: microwave-assisted freeze-drying (MFD) [13]. Here, it is specifically used for high-value goods like dry fruit [14]. Similar to the conventional freeze-drying process, the material to be dried first needs to be frozen. In a second step, the drying itself takes place. In contrast to CFD, the main heat transfer mechanism is radiation rather than convection and conduction. Especially polar substances, e.g., water, sugars, and amino acids, show good absorption of electromagnetic waves of wavelengths of 12.2 cm and frequencies of 2.45 GHz [15,16]. In brief, the heating mechanism in pharmaceutics occurs due to dipolar and ionic mechanisms. When such a polar compound is placed in an oscillating field, dipoles or ions try to realign in the direction of the electric field. Due to the ultra-rapid change in the direction of the electric field, internal friction of the molecules is caused, leading to heating within the material, i.e., volumetric heating. In the case of ions, a charge-driven migration is discussed [16,17,18]. MFD has clear advantages over conventional drying processes, like significantly shorter process times [19,20], and in the field of food processing, in the maintenance of shape, color, taste, odor, and texture [14,21,22,23]. In the transition area between food and pharmaceutical technology, MFD was used for the gentle drying of bacteria suspensions. Ambros et al. [19] investigated the survival rate and viability of different bacterial cultures. They found comparable survival rates of the investigated cultures produced by MFD compared to conventional freeze-drying but were able to shorten process times by up to 80%. The first usage in pharmaceutical freeze-drying was presented by Evans et al. [24] at the CPPR Freeze Drying of Pharmaceuticals & Biologics Conference in 2014, showing the general applicability to monoclonal antibodies and vaccine formulations. On this basis, a handful of international patents were filed claiming engineering- [25] or formulation-/process-focused [26,27] intellectual property. In a previously published work from our group [20], the general applicability to various pharmaceutical freeze-drying excipient systems containing a monoclonal antibody was underlined. Moreover, the potential for process drying time reductions was discussed. However, two major questions that have been raised have not been answered yet: (1) How do different microwave-assisted freeze-dried antibody formulations perform in accelerated stability studies with respect to solid-state and protein stability compared to a conventionally freeze-dried reference? (2) Is the inferior batch homogeneity found for MFD samples a general issue associated with microwave drying, or are there ways to improve it?

The current study examines four different formulations of two different monoclonal antibodies in the presence of three different glass-forming excipients: sucrose, trehalose, and arginine phosphate. These formulations were freeze-dried with two different drying protocols, i.e., using conventional freeze-drying and microwave-assisted freeze-drying. Moreover, a new microwave setup equipped with a semiconductor solid-state microwave generator was used for one of the formulations. Samples were stored for 24 weeks at different temperatures (2–8 °C and 40 °C) and analyzed at fixed times for their solid-state and protein-related quality attributes. We hypothesize that, on the one hand, irrespective of the monoclonal antibody formulation, comparable stability profiles can be found for CFD and MFD. On the other hand, we anticipate the new microwave machinery setup to have a positive effect on batch homogeneity in microwave-assisted freeze-dried products. 

## 2. Materials and Methods 

### 2.1. Materials

Two different IgG type 1 monoclonal antibodies (mAb) were investigated. mAb1 was kindly provided by Boehringer Ingelheim Pharma GmbH & Co. KG (Ingelheim am Rhein, Germany). mAb2 was an on stock at Ludwig-Maximilians-Universität München (LMU).

For mAb1-formulations, the following excipients were used: ACS certified D(+) Sucrose, which was purchased from Sigma-Aldrich (Steinheim, Germany), D(+) Trehalose dihydrate (min. 99% purity) was obtained from VWR International BVBA (Leuven, Belgium). EMPROVE^®^ exp L-Arginine (Ph. Eur. certified), EMSURE^®^ ortho-Phosphoric acid 85% and Ph. Eur. certified Tween 80^®^ were obtained from Merck KGaA (Darmstadt, Germany). For mAb2-formulation, EMPROVE^®^ exp sucrose (Ph.Eur.-certified) purchased from Merck KGaA (Darmstadt, Germany) was used.

L-Histidine monohydrochloride monohydrate (min. 99% purity) and L-Histidine (Cell culture reagent) were purchased from Alfa Aesar (Karlsruhe, Germany). Di-sodium hydrogen phosphate dihydrate and sodium dihydrogen phosphate dihydrate were obtained from AppliChem (Darmstadt, Germany). Trizma^®^ base BioXtra (>99.9%) and Trizma® hydrochloride BioXtra (>99.0%) were purchased from Sigma-Aldrich (St. Louis, MO, USA). Sodium chloride was obtained from Bernd Kraft (Duisburg, Germany). Sodium hydroxide was purchased from Merck KGaA (Darmstadt, Germany).

For the preparation of buffers and stock solutions, water for injection (WFI; Purelab Plus, USF Elga, Celle, Germany) was used. 

### 2.2. Study Design

The four different formulations, F1–F4, which were dried either by microwave-assisted freeze-drying (MFD) or by conventional freeze-drying (CFD), were stored for 24 weeks at refrigerator temperature 2–8 °C, at 25 °C (F1), and at 40 °C (Table 1). The low concentration mAb formulations (F1–F3) were produced using a previously described MFD setup with a 2 kW/2450 MHz magnetron [20], whereas the high concentration mAb formulation (F4) was processed using a novel semiconductor solid-state microwave radiation source tunable from 5 W to 450 W/2450 MHz. 

### 2.3. Preparation of Formulations

mAb1 was dialyzed against 10 mM histidine buffer (F1, F2) or 10 mM arginine phosphate (F3) at pH 6.0 for 24 h using dialysis membranes Spectra/Por^®^ (MWCO 6000–8000 Da; Spectrum Laboratories Inc., Compton, CA, USA) with two buffer exchanges. After dialysis, the concentration of mAb1 was measured with a NanoDrop™ 2000 UV photometer (Thermo Scientific, Wilmington, Delaware) at 280 nm using an extinction coefficient of ε_0.1%_ = 1.49 g/100 mL^−1^ cm^−1^. 

mAb2 (F4) was dialyzed and concentrated using a cross-flow filtration unit Minimate™ TFF capsule with omega polyethersulfone (PES) membrane (MWCO 30,000 Da; Pall Corporation, New York, NY, USA) by adding a 10-fold excess of 10 mM histidine buffer (pH 5.5). After reaching the desired volume, the concentration of the mAb was measured with a NanoDrop™ 2000 UV photometer at 280 nm using an extinction coefficient of ε = 225,000 M^−1^ cm^−1^ and a molecular weight of MW = 145.5 kDa.

Formulations were prepared according to the composition shown in Table 1.

F1–F3 were filtered using 0.2 µm PES membrane syringe filters (VWR International, Radnor, PA, USA), whereas F4 was filtered using a 0.22 µm PES Sartolab^®^ RF vacuum filter unit (Sartorius AG, Goettingen, Germany). For each formulation, 2.3 mL was filled in 10R tubing vials (MGlas AG, Muennerstadt, Germany) and semi-stoppered with lyophilization stoppers (FluroTec^®^ rubber stopper, West Pharmaceuticals, Eschweiler, Germany). The vial population for conventional freeze-drying was arranged on a lyophilization tray, and surrounded with at least one row of 10% (*w/v*) sucrose shielding vials.

### 2.4. Freeze-Drying Process

All samples of a corresponding formulation were frozen in the same freezing step. The formulations F1–F3 were frozen in a Christ ε2-6D laboratory-scale freeze-dryer (Martin Christ, Osterode am Harz, Germany) with equilibration at −5 °C for 1 h, followed by ramping down the shelf with 1 K/min to a −60 °C set point.

Formulation F4 was frozen in an FTS Systems LyoStar™ 3 freeze-dryer (SP Scientific, SP Scientific, Stone Ridge, NY, USA) with equilibration at 5 °C for 1 h, followed by ramping down the shelf with 1 K/min to a −50 °C set point.

The frozen samples were subjected to one of the following drying protocols:

#### 2.4.1. Conventional Freeze-Drying (CFD)

The conventional freeze-drying cycles are summarized in Table 2. The freeze-dryer used for cycle F1 and F2/F3 was not equipped with process analytical technologies for sublimation endpoint determination like comparative pressure measurement. Several different formulations were dried in one run at the same time. The holding time for primary drying, therefore, was chosen to allow for completed sublimation.

#### 2.4.2. Microwave-Assisted Freeze-Drying (MFD)

Microwave-assisted freeze-drying was conducted on a modified laboratory-scale Püschner µWaveVac 0250fd vacuum dryer prototype (Püschner GmbH + Co KG, Schwanewede, Germany). 

**Figure 1:**
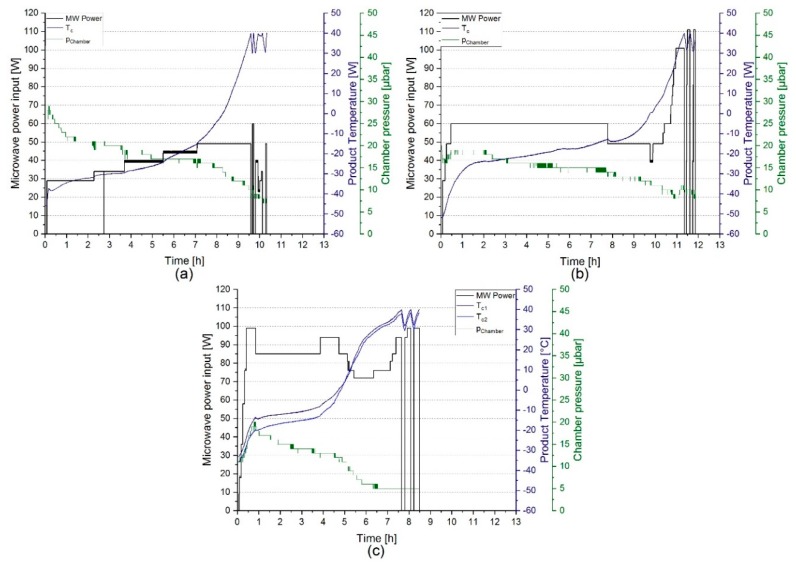
Graphical overview of the microwave-assisted freeze-drying (MFD) processes for (**a**) F1, (**b**) F2/F3, and (**c**) F4. Microwave power input (MW Power) is the actual measured radiated microwave power, the chamber pressure is the Pirani gauge readout (p_Chamber_), and Tc represents the readout of the glass fiber temperature probe.

For the formulations F1–F3, the setup described previously [20] was used. Briefly, it contained a 2 kW/2450 MHz magnetron, a condenser (−80 °C), and a vacuum system comprising a root pump and a rotary vane pump. The tuner, which was located between the magnetron and water load, was adjusted so that approximately 1/10 of the generated microwaves went into the product chamber. For a schematic overview of the general setup, the reader is referred to reference [28]. In the setup used for F1–F3, water load and product cavity were interchanged. Drying was carried out at a pressure of 8 µbar to 30 µbar measured by Pirani gauge, and at a microwave power between 23 W to 110 W measured by a HOMER™ impedance analyzer (S-TEAM Lab, Bratislava, Slovak Republic), until a constant mass was reached. The drying process used for F1 and F2/F3 is presented in Figure 1a,b, respectively. 

In contrast, F4 was processed with a partially modified setup comprising a semiconductor solid-state 500 W/2450 MHz microwave radiation source tunable from 5 W to 450 W, which directly emitted its radiation into the product chamber [28]. Moreover, the vacuum system was complemented by the addition of a turbopump to allow for lower chamber pressures. Drying, as it is shown in Figure 1c, was carried out at a pressure of 5 µbar to 20 µbar measured by Pirani gauge and at a microwave power between 18 W to 99 W measured by a HOMER™ impedance analyzer (S-TEAM Lab, Bratislava, Slovak Republic).

For process monitoring, two glass fiber temperature measurement probes (TS2, Weidmann Technologies Deutschland GmbH, Dresden, Germany) were used. Stoppering of the samples was carried out externally in a glove bag flushed with dry nitrogen. The dried crimped samples were kept refrigerated until analysis.

### 2.5. Karl Fischer Titration

Karl Fischer titration, equipped with a headspace module, was used to determine residual water content after freeze-drying. Between samples, aliquots of 9 mg and 28 mg were prepared in a glove box filled with pressurized air with a relative humidity of less than 10%, placed into 2R vials, and stoppered. The samples were then placed in an oven with 100 °C to enable the fast extraction of water. The headspace moisture is transported into a coulometric Karl Fischer titrator (Aqua 40.00, Elektrochemie Halle, Halle (Saale), Germany). Results were calculated in relative water content (*w/w*). For verification of equipment performance, three aliquots of Apura^®^ water standard oven 1% by Merck KGaA (Darmstadt, Germany) were measured within a sequence.

### 2.6. Brunauer-Emmet-Teller Krypton Gas Adsorption

The specific surface area of dried samples was determined using Brunauer–Emmet–Teller (BET) krypton gas adsorption in a liquid nitrogen bath at 77.3 K (Autosorb 1; Quantachrome, Odelzhausen, Germany). Samples of 90–200 mg were gently crushed with a spatula and weighed into glass tubes. Prior to measurement, an outgassing step was performed for at least 6 h at room temperature. An 11-point gas adsorption curve was measured, covering a p/p_0_ ratio of approximately 0.05–0.30. Data evaluation was performed according to the multi-point BET method fit of the Autosorb 1 software.

### 2.7. X-Ray Powder Diffraction 

To determine the solid-state of the lyophilizates, an XRD 3000 TT diffractometer (Seifert, Ahrensburg, Germany) was used. The device was equipped with a copper anode (40 kV, 30 mA) and had a wavelength of 0.154178 nm. The scintillation detector voltage was 1000 V. The samples were placed on the copper sample holder and analyzed in the range of 5–45° 2-theta, with steps of 0.05°.

### 2.8. Reconstitution of Lyophilizates

The lyophilized cakes were reconstituted by the addition of WFI. The WFI volume for each formulation was calculated to match the volume of the water removed during freeze-drying. Reconstitution time was determined by recording the time between adding the respective formulation-specific volume of water for injection and obtaining a clear solution without visible matter. This observation was performed by manual visual inspection. Reconstitution was performed applying gentle swirling for 5 s directly after the addition of water.

### 2.9. High-Performance Size Exclusion Chromatography (HP-SEC)

A Waters 2695 Separation module (Waters GmbH, Eschborn, Germany) equipped with a Waters 2487 Dual λ Absorbance Detector (Waters GmbH, Eschborn, Germany) at 214 and 280 nm was used. Isocratic elution with a 25 mM sodium phosphate running buffer containing 200 mM sodium chloride (pH 7.0) was performed. 

For mAb1-formulations (F1–F3), 10 µL of a reconstituted solution corresponding to a loading of 50 µg protein were loaded on a Tosoh TSKgel G3000SWxl, 7.8 × 300 mm, 5 µm column (Tosoh Bioscience, Griesheim, Germany) and separated with a flow rate of 0.7 mL/min. 

For mAb2 (F4), samples were diluted with 10 mM histidine buffer (pH 5.5) to 1 g/L protein concentration, and 25 µL was injected, corresponding to a load of 25 µg protein. A YMC-Pack Diol-300, 300 × 8.0 mm, 5 µm column (YMC Europe GmbH, Dinslaken, Germany) with a flow rate of 0.8 mL/min was used for separation. Samples were measured in triplicates with three individual injections. Data integration of relative areas at 280 nm was performed using Chromeleon 6.80 (Thermo Scientific, Wilmington, DE, USA), provided that every peak eluting before the monomer corresponded to high molecular weight (HMW) species. No peaks could be detected after the monomer. For verification of equipment performance, an internal standard of thawed mAb formulation was injected at the beginning and end of a sequence. 

### 2.10. High-Performance Cation Exchange Chromatography (HP-CEX)

A Waters 2695 Separation module (Waters GmbH, Eschborn, Germany) equipped with a Waters 2487 Dual λ Absorbance Detector (Waters GmbH, Eschborn, Germany) at 214 and 280 nm was used for weak cation exchange chromatography. A linear sodium chloride gradient of 0% to 20% solvent B in solvent A over 30 min was used for elution at a flow rate of 1 mL/min. For all cation exchange (CEX) analysis, a ProPac™ WCX-10G BioLC™ Analytical column 4 × 250 mm equipped with a ProPac™ WCX-10G BioLC™ guard column 4 × 50 mm (ThermoFisher Scientific, Waltham, MA, USA) was used. 

For mAb1-formulations (F1–F3), the solvents were composed of A: 20 mM TRIS (pH 7.1) and B: 20 mM TRIS (pH 7.1) plus 300 mM sodium chloride. Reconstituted sample aliquots of 10 µL, corresponding to a loading of 50 µg protein, were loaded on the column. 

For mAb2 (F4), the solvents were composed of A: 20 mM TRIS (pH 7.5) and B: 20 mM TRIS (pH 7.5) plus 300 mM sodium chloride. Before analysis, samples were diluted with solvent A to 1 g/L protein concentration, and 50 µL was injected, corresponding to a load of 50 µg protein. 

Samples were measured as triplicates with two individual injections. Data integration of relative areas was performed using the Chromeleon 6.80 software (Thermo Scientific, Wilmington, DE, USA), provided that every peak eluting before the main peak corresponded to acidic species and peaks eluting after the main peak corresponded to basic species. For verification of equipment performance, an internal standard of thawed mAb formulation was injected at the beginning and end of a sequence.

### 2.11. Light Obscuration

One method used to determine subvisible particles of the formulation F1 was light obscuration. Therefore, a PAMAS SVSS-35 particle counter (PAMAS—Partikelmess- und Analysesysteme GmbH, Rutesheim, Germany) equipped with an HCB-LD 25/25 sensor, which had a detection limit of approximately 120,000 particles ≥ 1 µm per mL, was used. The pre-rinsing volume was 0.4 mL and was followed by three measurements of 0.2 mL. The fill rate, emptying rate, and rinse rate of the syringe were set to 10 mL/min. Before and between samples, the system was rinsed with WFI until less than 30 particles/mL ≥ 1 µm and no particles larger than 10 µm were present. Data collection was done using PAMAS PMA software, and particle diameters in the range of ≥1 µm to 200 µm were determined. All results are given in cumulative particles per milliliter.

### 2.12. Flow-Imaging Microscopy

Due to the high transparency of protein particles, an orthogonal method for subvisible particle determination was introduced for formulations F2–F4. Flow-imaging microscopy was performed on a FlowCAM^®^ 8100 (Fluid Imaging Technologies, Inc., Scarborough, ME, USA) equipped with a 10× magnification cell (81 µm × 700 µm). Prior to a measurement set, the cell was cleaned with a 1% Hellmanex III solution and WFI. For adjustment of the focus, the default autofocus procedure using 20 µm calibration beads was performed. Sample solution volumes of 150 µL were measured with a flow rate of 0.10 mL/min, at an image rate of 29 frames per second, and an estimated run time of 1.5 min. After each measurement, the flow cell was flushed with WFI. For particle identification, the following settings were used: 3 µm distance to the nearest neighbor, particle segmentation thresholds for dark pixels and light pixels of 13 and 10, respectively. The particle size was reported as the equivalent spherical diameter (ESD). Frames were collected with VisualSpreadsheet^®^ 4.7.6 software and were evaluated for total particle counts of cumulative particles greater or equal to 1 µm, 10 µm and, 25 µm per mL.

## 3. Results

### 3.1. Solid State

#### 3.1.1. Residual Moisture Content and Specific Surface Area

**Figure 2:**
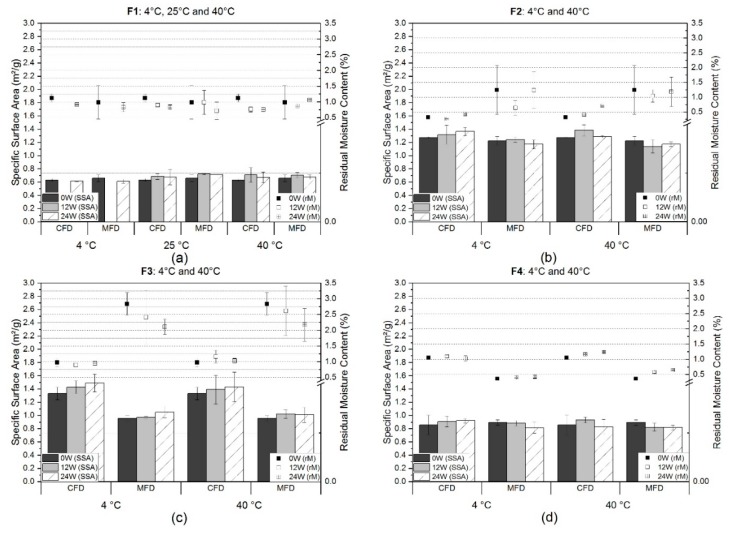
Specific surface area (bars) and residual moisture content (squares) results over the course of 24 weeks of storage at the respective storage temperature for (**a**) F1, (**b**) F2, (**c**) F3, and (**d**) F4. Values shown represent the mean value (*n* = 3) ± standard deviation.

The results for residual moisture (RM) and specific surface area (SSA) determination are presented in Figure 2. For the low concentrated mAb formulation with sucrose (F1), three different storage temperatures are shown (Figure 2a). Directly after freeze-drying, the SSA results revealed identical values for CFD, 0.63 m^2^/g ± 0.02 m^2^/g, and MFD, 0.66 m^2^/g ± 0.05 m^2^/g. Irrespective of the storage temperature and time point, neither differences nor relevant changes in specific surface areas were observed. With regard to residual moisture content, analysis showed low values for both CFD and MFD (1.1% ± 0.1% and 1.0% ± 0.5%, respectively). These values changed only slightly over 24 weeks at all investigated storage temperatures. However, within the microwave-processed products, some samples exhibited higher variances represented by higher standard deviations, which were not found within the conventionally freeze-dried samples. 

Similar results were found for the low concentrated mAb formulation stabilized with trehalose (Figure 2b). No relevant differences or changes were observed for the specific surface area over the course of 24 weeks storage at refrigerator temperature or 40 °C. Mean values almost remained at initial values of 1.27 m^2^/g ± 0.01 m^2^/g and 1.22 m^2^/g ± 0.06 m^2^/g for CFD and MFD, respectively. In regards to residual moisture, MFD samples appeared to be moister than CFD samples (1.2% ± 0.8% vs. 0.3% ± 0.0%). These differences remained over the course of six months of storage irrespective of the storage temperature. Yet, the moisture content in CFD cakes doubled at 40°C (0.7% ± 0.0%), unlike MFD cakes. However, high variances within MFD samples may have masked such effects.

Unlike the sucrose (F1) and trehalose (F2) formulations, low concentration mAb formulations with arginine phosphate (F3) exhibited differences with respect to specific surface area (Figure 2c). Initial measurements directly after freeze-drying revealed values of 1.33 m^2^/g ± 0.09 m^2^/g and 0.95 m^2^/g ± 0.04 m^2^/g for CFD and MFD, respectively. Slight, but non-significant changes over storage were observed. The initially different SSA values correlated inversely with the observed residual moisture mean values (CFD: 1.0% ± 0.1% and MFD: 2.8% ± 0.4%). A micro-collapse within MFD samples was assumed. 

F4, which was comprised of a 1:1-mixture (weight-wise) of sucrose and mAb2, was dried with a different microwave-setup. By this, high variances in residual moisture, which occasionally occurred before within MFD samples, were not observed anymore (Figure 2d). For conventional freeze-dried samples, mean values changed from 1.0% (±0.0%) to 1.2% (±0.0%) over 24 weeks of storage at 40 °C. Within MFD samples, a similar increase from 0.4% (±0.0%), initially to 0.6% (±0.0%), was observed after six months at 40 °C. In contrast, specific surface areas were found to remain unaffected by accelerated storage conditions at values of 0.85 m^2^/g ± 0.15 m^2^/g and 0.89 m^2^/g ± 0.04 m^2^/g for CFD and MFD, respectively. 

#### 3.1.2. X-Ray Powder Diffraction (XRD) 

In order to confirm the amorphicity of all formulations, XRD was used. The results for the two sucrose-based formulations, F1 and F4, are presented in Figure S1 and were directly compared to the pure excipient sucrose. No indications of crystallization were found. 

Amorphous halos and the absence of typical peaks [29,30] were found for trehalose-based formulations (Figure S2).

An overall XRD-amorphicity, represented by an amorphous halo, was also found for the significantly moister arginine phosphate formulations (Figure S3). A reference diffractogram for recrystallized arginine phosphate was derived by intentionally exposing one MFD vial to a moist atmosphere overnight.

### 3.2. Protein-Related Quality Attributes

#### 3.2.1. Reconstitution and Subvisible Particles (SvP)

Before liquid analysis, lyophilized products needed to be reconstituted. Within one formulation, no significant difference between the distinct drying protocols was seen. However, small differences with regard to other formulation were observed (Table 3).

**Figure 3:**
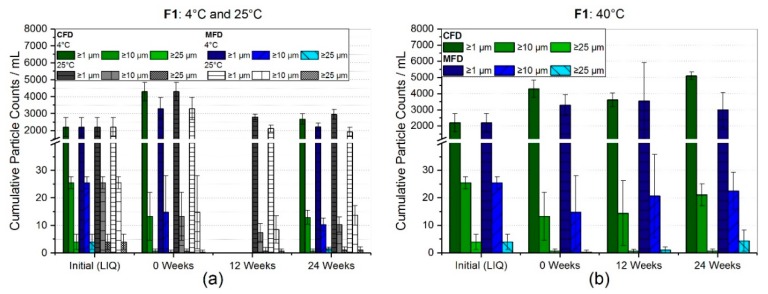
Subvisible particle (SvP) counts for formulation F1 measured by light obscuration and size-exclusion chromatography results. The bar charts represent the subvisible particle counts for the respective storage temperatures (**a**) 4 °C, 25 °C, and (**b**) 40 °C. Bars represent the mean value ± standard deviation for three individual vials.

The subvisible particle counts (SvP) obtained by light obscuration for F1 are presented in Figure 3a,b. All vials analyzed originated from the same filtered bulk formulation, which is why initial particle counts were the same before a certain drying or storage scheme was applied. Light obscuration measurements revealed relatively low particle counts per mL of 2212 ± 565, 26 ± 2 ,and 4 ± 3 for ≥1 µm, ≥10 µm, and, ≥25 µm, respectively. An increase of +94% (4300 ± 546) and +49% (3303 ± 651) for cumulative particles ≥1 µm/mL was observed directly after freeze-drying. In Figure 3a the results after storage over 24 weeks at 4 °C and 25 °C are shown. After six months at refrigerator temperature, particle counts were stabilized close to values prior to freeze-drying of 2678 ± 307 (CFD) and 2227 ± 225 for particles ≥1 µm/mL. At 25°C, storage temperature, subvisible particle counts were only slightly elevated for CFD (2953 ± 295), but not for MFD (1937 ± 247). No increase in bigger particles, ≥10 µm, and ≥25 µm, was seen at any of the storage conditions. Figure 3b shows that storage over 24 weeks at 40 °C caused an increase by factor 2.3 (5106 ± 237) for conventionally FD, and an increase of 36% (3003 ± 1058) for microwave-assisted FD, in ≥1 µm particles. However, bigger variances were found for MFD samples. 

**Figure 4:**
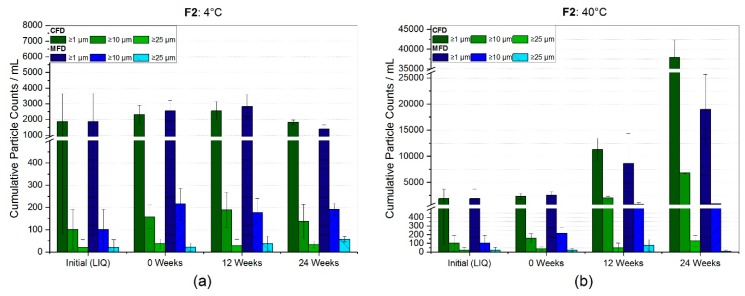
Subvisible particle (SvP) counts for trehalose-based formulation F2 measured by flow-imaging microscopy. Bar chart (**a**) represents the SvP counts at refrigerator storage and (**b**) at 40 °C. Bars represent the mean value ± standard deviation for three individual vials.

Figure 4 represents the SvP counts analyzed by flow-imaging microscopy for F2. The same filtered bulk formulation was used for all vials analyzed. Initially, relatively low particle counts per mL of 1867 ± 1784, 90 ± 20 and 20 ± 35 for ≥1 µm, ≥10 µm, and ≥25 µm, respectively, were found. A slight increase by 24% (2320 ± 599) and 37% (2549 ± 677) for cumulative particles ≥1µm/mL was observed directly after freeze-drying. At 4 °C storage temperature, particle numbers ≥1 µm/mL settled around initial values after 24 weeks, but the cumulative count of bigger particles increased (Figure 4a). However, no significant changes were observed. A dramatic increase for SvP ≥1 µm/mL was found over the course of six months at 40°C for both CFD (37909 ± 4337) and MFD (18947 ± 6753), as shown in Figure 4b. The mean SvP count values for ≥10 µm and ≥25 µm also increased drastically, even though high standard deviations lowered the significance. However, an upward trend could be assumed for subvisible particles ≥10 µm/mL, in conventionally processed samples.

**Figure 5:**
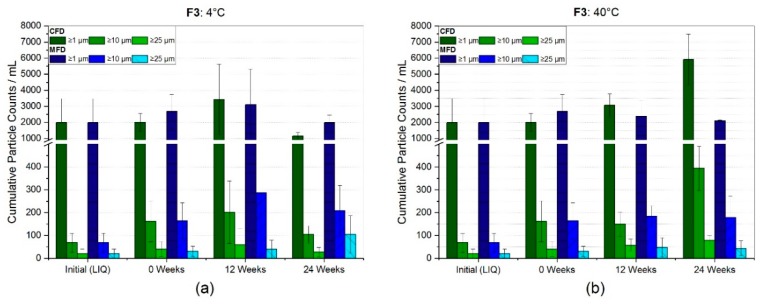
Subvisible particle (SvP) counts for the arginine phosphate formulation F3 measured by flow-imaging microscopy. Bar chart (**a**) represents the SvP counts at refrigerator storage and (**b**) at 40 °C. Bars represent the mean value ± standard deviation for three individual vials.

The SvP counts for the low concentration mAb formulation stabilized by arginine phosphate, F3, are shown in Figure 5. Prior to freeze-drying, relatively low particle counts per mL of 1991 ± 1490, 68 ± 42 and 20 ± 21 for ≥1 µm, ≥10 µm, and ≥25 µm were found, respectively. At 4 °C (Figure 5a) storage temperature, a small increase in mean values for cumulative particles ≥1 µm was found over time, although particle counts for this size category settled around the initial values. For particles ≥10 µm and ≥25 µm, a slightly stronger increase in mean values was observed, although vast standard deviations lowered the significance. 

At accelerated storage conditions (Figure 5b), a moderate increase in all size categories was seen. Especially after six months storage, the conventionally freeze-dried sample showed a significant increase for ≥1 µm, ≥10 µm, and ≥25 µm with particle counts per mL of 5913 ± 1584 (3× higher), 394 ± 97 (6× higher), and 78 ± 21 (4× higher), respectively. In contrast, the microwave-assisted freeze-dried sample at the same conditions showed no increase for ≥1 µm, and only a modest increase by factor 2.6 and 2.2 for cumulative particle counts ≥10 µm and ≥25 µm, respectively.

**Figure 6:**
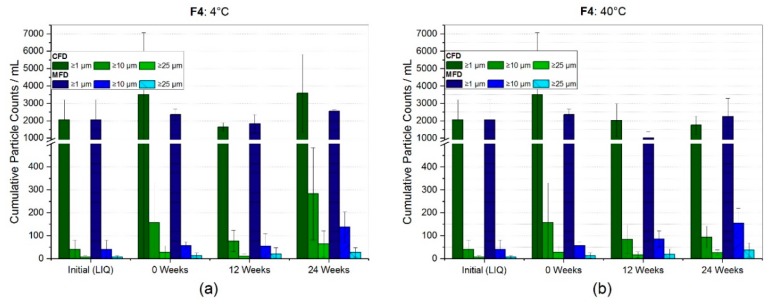
Subvisible particle (SvP) counts for the high concentration mAb formulation with 50 g/L stabilized by sucrose, measured by flow-imaging microscopy. Bar chart (**a**) represents the SvP counts at refrigerator storage and (**b**) at 40 °C. Bars represent the mean value ± standard deviation for three individual vials.

For the formulation with 50 g/L of mAb2 and only 5% (*w/v*), sucrose-stabilizer neither at refrigerator (Figure 6a) nor at 40 °C storage temperature (Figure 6b), was a significant change in subvisible particles observed. The initial formulation before FD revealed low particle counts per mL of 2051 ± 1153, 40 ± 41 and 7 ± 6 for ≥1 µm, ≥10 µm, and ≥25 µm, respectively. The results obtained showed a larger increase by 74% (3579 ± 2243) for CFD and 25% (2555 ± 97) for MFD, in regards to cumulative particles ≥1 µm at four degrees celsius storage over six months, compared to storage at 40 °C. Yet, at all conditions observed, larger particle categories, i.e., ≥10 µm and ≥25 µm, revealed an increased number of particle counts. However, due to high standard deviations within a sample, no significant changes were detectable.

#### 3.2.2. Weak Cation Exchange Chromatography (CEX)

**Figure 7:**
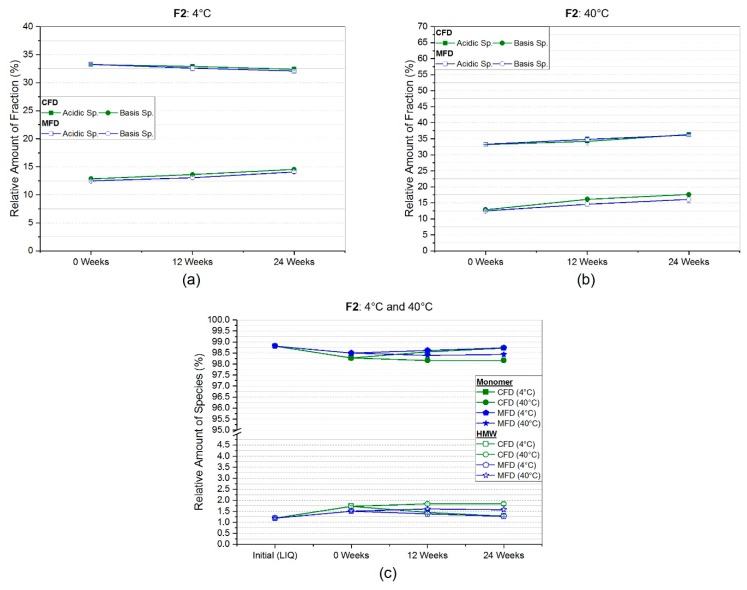
Relative amount of acidic and basic species obtained by high-performance (HP)-weak cation exchange chromatography for trehalose-based formulation F2 at (**a**) 4 °C and (**b**) 40 °C storage temperature. In (**c**), the relative percentages of monomer and high molecular weight species (HMW) at the respective storage temperature over storage time gained by HP-size exclusion chromatography (SEC) analysis are presented.

In order to generically quantify different protein degradation pathways, e.g., deamidation, a salt-gradient weak cation exchange chromatography was used. The CEX data for F1 was not collected. But for the trehalose-based low concentration mAb formulation (F2), data from CEX measurements is shown in Figure 7a,b for the respective storage temperatures. Directly after freeze-drying, irrespective of the applied drying protocol (CFD or MFD), relative amounts of the different species were found to be alike. For storage at refrigerator temperature, (Figure 7a) acidic species dropped by roughly one percent for both drying protocols, whereas basic species slightly increased by 1.5%. Somewhat more pronounced changes were observed for storage at 40 °C (Figure 7b). While acidic species increased by three percent, basic species rose by 4.7% and 3.5% for CFD and MFD, respectively. 

**Figure 8:**
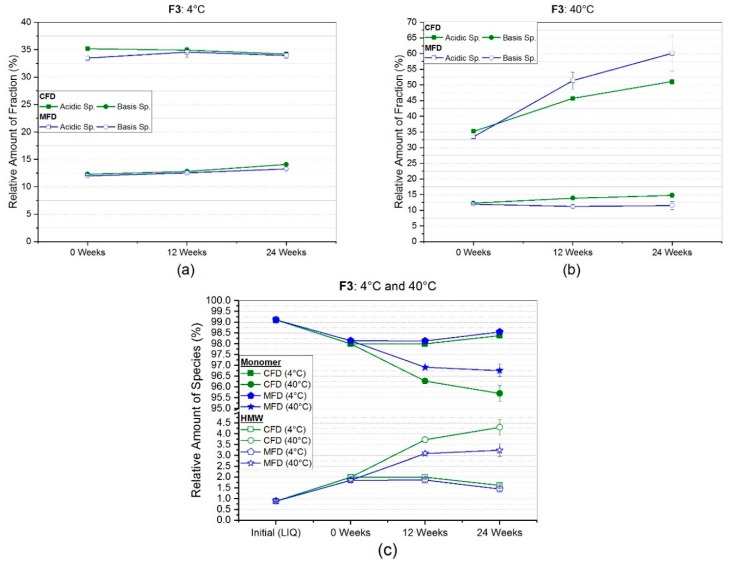
Relative amount of acidic and basic species obtained by HP-weak cation exchange chromatography for arginine phosphate-based formulation F3 at (**a**) 4 °C and (**b**) 40 °C storage temperature. In (**c**), the relative percentages of monomer and high molecular weight species (HMW) at the respective storage temperature over storage time gained by HP-SEC analysis are presented.

For the arginine phosphate-formulation (F3), CEX data is shown in Figure 8a,b. A small difference of 1.7% in the initial relative amount of acidic species was found, which equalized for the two drying protocols over 24 weeks storage time at 4 °C (Figure 8a). At the same conditions, basic species slightly increased for both CFD (+1.8%) and MFD (+1.3%), revealing a small deviation. Acidic species showed a noticeable change by 15.9% (CFD) and 26.6% (MFD) at 40 °C storage temperature (Figure 8b), whereas basic species remained almost the same with changes by 2.5% (CFD) and −0.5% (MFD). 

**Figure 9:**
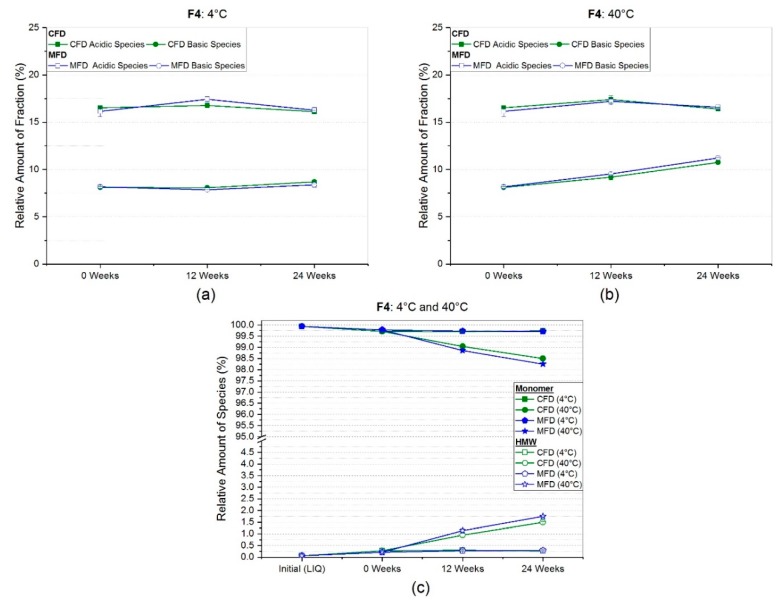
Relative amount of acidic and basic species obtained by HP-weak cation exchange chromatography for arginine phosphate-based formulation F3 at (**a**) 4 °C and (**b**) 40 °C storage temperature. In (**c**), the relative percentages of monomer and high molecular weight species (HMW) at the respective storage temperature over storage time gained by HP-SEC analysis are presented.

In the case of the high concentration mAb formulation stabilized with sucrose (F4), almost no differences were found between conventional and microwave-assisted freeze-dried products with regard to CEX results (Figure 9a,b). At refrigerator temperature (Figure 9a), changes in both species and both drying protocols ranged within less than 0.5%. While similar observations were made for the acidic species at 40 °C, basic species slightly rose by 2.7% and 3% for CFD and MFD samples, respectively. However, no difference between the drying procedures was observed. 

#### 3.2.3. Size Exclusion Chromatography (SEC)

The relative amount of monomeric and high molecular weight (HMW) species was assessed by high-performance size-exclusion chromatography. The results of the HP-SEC analysis for the low concentration mAb formulation with 10% (*w/v*) sucrose (F1) are displayed in Figure S4. Irrespective of the storage temperature, no changes in monomer content occurred. In other words, the relative amount of monomeric species ranged between 99.0% to 99.1% at all analyzed time points, and all investigated storage temperatures. 

Only a slightly different picture was seen for the trehalose formulation F2 in Figure 7c. After six months at 4 °C, the loss of monomer and the complementary rise in HMW was negligibly low, basically within the range of SEC sample standard deviation. A small decrease by −0.6% (98.2% ± 0.1%) for CFD and by −0.4% (98.4% ± 0.1%) for microwave-assisted freeze-dried lyophilizates was observed in accelerated storage conditions of 40 °C for 24 weeks.

In arginine phosphate-based monoclonal antibody formulation (F3), more changes were observed (Figure 8c). At refrigerator temperature, a small decrease by less than one percent in monomer content, and thus, an increase in HMW aggregates to less than 2% overall HMW species, occurred. However, a significant loss in monomeric content down to 95.7% ± 0.4% and 96.8% ± 0.3% for CFD and MFD, respectively, was seen. This was counterbalanced by an increase in high molecular weight species to 4.3% ± 0.4% and 3.2% ± 0.3% for conventionally and microwave-assisted freeze-dried samples.

The formulation with 50 g/L of mAb2 (F4) showed a higher monomeric SEC-purity of 99.9% ± 0.0%, compared to mAb1 formulations prior to freeze-drying (Figure 9c). At refrigerator storage temperatures, only a small loss of 0.2% relative monomer content was observable regardless of the initially used drying procedure. Even for 24 weeks at 40 °C, the monomer content for CFD and MFD samples decreased only slightly to 98.5% ± 0.0% and 98.2% ± 0.0%, respectively. This loss was compensated by an increase in HMW to 1.5% ± 0.0% and 1.8% ± 0.0% for conventionally and microwave-assisted freeze-dried samples, respectively. 

## 4. Discussion

### 4.1. Stability with Regard to Solid State Properties

In the case of freeze-dried products, attributes like residual moisture content, solid-state, and specific surface area are critical to assess and to monitor over storage and shelf-life [31]. With respect to the specific surface area, the low mAb concentration formulations with sucrose and trehalose, F1 and F2 (Table 1), respectively, showed identical values for each formulation for the two distinct drying protocols directly after freeze-drying, and showed no change over the duration of storage. This strongly indicated the absence of a microscopic collapse, which means that the initially determined ice and successive pore structure remained during the two different drying protocols for respective formulations [32]. Regarding the residual moisture content similar mean values were found for the sucrose-based formulation F1, at 1.1% ± 0.1% and 1.0% ± 0.5% for CFD and MFD, respectively (Figure 2a). This moisture level was kept over the storage duration, even at elevated temperatures. However, high variances in some MFD samples were observed. Such high variances among samples of one microwave-batch may have been caused by non-uniform temperature distribution. This non-uniformity in microwave heating was reported in the literature to be one major challenge associated with that drying technique [15,18,33]. The resulting appearance of cold and hot spots was described as multifactorial and may be dependent on the chosen mode (multimode, single-mode) [15,34], oven design [33], sample composition, geometry of the frozen good [15,35,36,37], occurrence of standing wave effect [38], and drying duration [39].

In contrast, in trehalose samples (F2), residual moisture levels differ already after the freeze-drying step, i.e., 0.3% ± 0.0% for conventionally dried and 1.2% ± 0.8% for microwave-assisted dried samples (Figure 2b). The CFD samples stored at 40 °C showed an increase to 0.7% ± 0.0%. This was most likely due to moisture uptake of the cake from the rubber stopper, as was described by Pikal and Shah [40]. The equilibration of stopper moisture and cake moisture was found to be kinetically dependent on storage temperature in the first place. Because of the high variances in MFD samples, such an effect may have happened but could not be detected. 

An arginine phosphate-based formulation (F3) was expected to be different. Firstly, because of the permanently charged character of arginine which causes high dielectric loss, i.e., the ability of a material to absorb electromagnetic energy [15], as reported by Meng et al. [41]. Of course, the dielectric properties of a material may vary depending on the exact composition, density, temperature, and frequency [15]. Nevertheless, different behavior of such formulation within the electromagnetic microwave field was expected. 

Secondly, because of the reported complex molecular structure of arginine phosphate [42]. Although the reported structure was described for the crystalline state, similar intense interactions in the glassy state were assumed [43,44]. For formulation F3 specific surface area values were reduced by 39% to 0.95 m^2^/g ± 0.04 m^2^/g for MFD, compared to 1.33 m^2^/g ± 0.09 m^2^/g for CFD, initially after application of the different drying protocols (Figure 2c). It is assumed that this shift in the SSA is associated with a microscopic collapse within the amorphous matrix, which may have been favored by the permanently charged matrix leading to enhanced absorption of microwave energy [41]. As the desorption step of the unfrozen water is highly SSA-dependent [45], the micro-collapse seems likely to be the cause for higher mean residual moisture in MFD (2.8% ± 0.4%), compared to CFD (1.0% ± 0.1%). Over the course of storage, no significant change in glassy state properties was observed.

The 50 g/L mAb2 formulation stabilized with sucrose (F4) appeared to be different with respect to residual moisture content (Figure 2d). The occasionally emerging high variances within one sample (*n* = 3) produced by MFD was not observed anymore. Two possible reasons are: 1) The formulation, which consisted of roughly 50% monoclonal antibody, may have changed the matrix properties; and 2) the newly implemented microwave technical setup as described in the materials and method section. The authors assume that the increase in batch homogeneity within MFD samples was mainly due to the change in machinery setup. This is believed because: Firstly, similar formulations with the same mAb have been dried with the previously described MFD setup [20], and they revealed the same high deviations that have been observed with the low concentrated mAb formulations F1–F3 in this study (Table S1). 

Secondly, with typical sample sizes of *n* = 3, potential batch inhomogeneities were observable. Thirdly, the solid-state semiconductor setup led to a more stable and better tuneable power input during the course of drying. Supportively, Bianchi et al. [46] simulated the physical behavior of apple slices under microwave-assisted vacuum drying processing comparing magnetron and solid-state technology. They concluded that with the latter, an improved heating pattern uniformity could be achieved.

With regard to X-ray diffraction analysis, all formulations revealed an XRD-amorphous solid-state exhibiting an amorphous halo (Figures S1–S3). By that, the authors suppose no adverse effect of microwaves on the crystallization tendency of the investigated matrices. Even moister samples have not revealed any indication of recrystallization.

### 4.2. Stability with Regard to Protein-Related Properties

The sucrose-based formulation with 5 g/L of mAb1 (F1) showed no clear trend in subvisible particles (LO) at 4 °C and 25 °C storage temperature (Figure 3a) and no difference between CFD and MFD either. Only directly after the freeze-drying procedure an increase of 94% (4300 ± 546) and 49% (3303 ± 651) for cumulative particles ≥1 µm/mL for CFD and MFD, respectively, was observed. Figure 3b shows that storage over 24 weeks at 40 °C caused an increase by factor 2.3 (5106 ± 237) for conventionally FD and an increase of 36% (3003 ± 1058) for microwave-assisted FD in ≥1 µm particles. It was expected that this slight change in SvP at accelerated storage conditions might correlate with an increase in the relative amount of high molecular weight species assessed by HP-SEC. However, no such effect on SEC data was seen (Figure S4). Across all storage temperatures and regardless of the drying protocol used, no change in neither soluble aggregates nor loss of monomer was observed. 

For the trehalose-based formulation (F2), the more sensitive flow-imaging microscopy technique was used for subvisible particle determination (Figure 4). Initially, low particle counts per mL of 1867 ± 1784, 90 ± 20 and 20 ± 35 for ≥1 µm, ≥10 µm, and ≥25 µm, respectively, were found, but yet bearing unusually high variances. Directly after freeze-drying, most likely due to freeze-drying associated stresses [47], a slight increase by 24% (2320 ± 599) and 37% (2549 ± 677) for cumulative particles ≥1 µm/mL was observed. No significant changes were observed for SvP at 4 °C storage temperature (Figure 4a). Taking chromatographic results into account, no HP-CEX (Figure 7a,b) or HP-SEC (Figure 7c) changes were observed, emphasizing sufficient and comparable stabilization in both drying populations at 4 °C. In contrary, a dramatic increase for subvisible particles ≥1 µm/mL was found over the course of six months at 40 °C, for both CFD (37,909 ± 4337) and MFD (18,947 ± 6753), as shown in Figure 4b. A similarly pronounced increase was also for bigger particles observable, although high standard deviations lowered significance. Yet, an upward trend was assumed for subvisible particles ≥10 µm/mL in conventionally processed samples. With respect to chemical degradation (Figure 7b), a linear increase with shallow slope was found for both acidic and basic species irrespective of the drying procedure, although basic species increased slightly more in CFD samples. An increase in the different species could be related to several different changes within the protein molecule depending on primary structure, cell line, formulation conditions, and so forth [48,49,50]. Reviewed by Du et al. [48] in 2012, a major contribution to acidic species was associated with the deamidation reaction of asparagine residues. For basic species, depending on primary structure, they discussed different causes covering C-terminal basic amino residues, including incomplete cyclization of the N-terminal, but also the formation of aggregates. It could be suspected that the stronger increase in high molecular weight aggregates (Figure 7c) at 40 °C in CFD samples is related to the stronger rise of basic species in HP-CEX data. However, no follow-up investigation by (partial) protein digestion or by LC-MS was conducted. Nonetheless, as differences between conventionally dried and microwave-assisted freeze-dried samples were rather marginal, comparable stability in the trehalose formulation was deduced.

Arginine phosphate, as discussed in Section 4.1, was expected to be an exceptional formulation, especially challenging when drying with electromagnetic waves. In a recently published review by Stärtzel [51], several examples of successful protein stabilization by arginine salts in the glassy state were shown. Within our study, we found only tiny changes at refrigerator temperature in regards to subvisible particles (Figure 5a). Primarily the mean values for bigger sized particles (≥10 µm and ≥25) increased after 24 weeks of storage, yet were not overly due to higher variances. HP-CEX data (Figure 8a) and HP-SEC data (Figure 8c) supported this. Only a slight degradation shown by relative monomer loss of less than 1% in both CFD and MFD, and a small increase in basic species of less than 2%, were found at 4 °C. In accelerated storage conditions (Figure 5b), a moderate increase in all size categories was seen. The conventionally freeze-dried sample especially showed a significant increase for cumulative particle counts ≥1 µm, ≥10 µm and, ≥25 µm, with factors of 3× to 6× higher counts after six months of storage. In contrast, the microwave-assisted freeze-dried sample at the same conditions showed only a moderate increase by bigger particles ≥10 µm and ≥25 µm. Connecting that to the HP-CEX data (Figure 8b), a contrary picture can be drawn. One the one hand, a noticeable change in acidic species by 15.9% (CFD) and 26.6% (MFD) at 40 °C storage temperature was found, whereas basic species remained almost the same for both drying procedures. On the other hand, HP-SEC data (Figure 8c) revealed a loss in monomeric content down to 95.7% ± 0.4% and 96.8% ± 0.3% for CFD and MFD, respectively. Taking the above into account, it appeared that the conventionally dried sample showed a higher degree of degradation, exhibiting a higher increase in high molecular weight aggregates, accompanied by an increased particle count in flow-imaging microscopy. However, HP-CEX results indicated that a moderate portion of the main charge variant underwent chemical reactions leading to significantly increased acidic species, being more pronounced in MFD samples. Stärtzel et al. [44] investigated different arginine salts on their stabilizing potential in the glassy state. For that, they also calculated the relaxation time τ^β^, “which may be regarded as proportional to the inverse of molecular mobility for global motions” [52]. Subsequently, they related physical aggregation rate constants at 40 °C with the estimated ln(τ^β^). They found that an arginine phosphate formulation (64 g/L L-Arg, 16 g/L sucrose and 50 g/L mAb) revealed longer relaxation times than other arginine formulations. These relaxation times unexpectedly had an inversely proportional correlation to the observed aggregation constants, which suggested that increased molecular mobility had a positive effect on protein stability [44]. This may be one explanation for the stability differences observed between CFD and MFD. Microwave-assisted freeze-dried samples on average, revealed higher residual moisture content (Figure 2c), compared to CFD. Residual water is known as a plasticizer of amorphous matrices, which also consequently leads to increased molecular mobility [53], and therefore potentially to reduced aggregation in an arginine-based system as reported by [44]. However, increased molecular mobility may be associated with increased chemical degradation [54,55], giving a potential explanation to the more distinct increase in acidic species MFD samples. Another explanation could be based on an advantageous effect of the partial collapse in MFD samples, as it was observed by Schersch et al. [56], for partially collapsed mannitol-sucrose formulations. All in all, microwave-assisted lyophilizates with arginine phosphate, on the one hand, revealed an indication for more pronounced chemical degradation, but on the other hand, showed a less severe increase in subvisible particles and aggregates. The authors, therefore, conclude a comparable stability profile for CFD and MFD with reservations. 

For the high concentration mAb2 formulation (F4), no clear trend could be derived from subvisible particle analysis. Unexpectedly, particle counts at refrigerator temperature appeared to be higher or at similar levels compared to 40 °C after six months (Figure 6). With regard to HP-CEX results, no difference between conventionally and microwave-assisted freeze-dried samples was observed (Figure 9a,b). In size-exclusion chromatography, samples stored at 4 °C exhibited a negligibly small loss of monomer for both drying protocols. At accelerated conditions, a rather low loss in monomeric species of 1.4% and 1.7% for CFD and MFD, respectively, was seen after six months of storage. For this reason, the authors conclude that both sample populations derived from the two respective drying protocols were comparable with respect to protein stability.

In the future, an improved prototype dryer with a sophisticated technical setup that provides the operator with standard pharmaceutical freeze-drying features, such as freezing and stoppering within the same machine, is needed. A combination of current pharmaceutical freeze-drying equipment with modern semiconductor solid-state microwave generators is imaginable, in the authors’ opinion. For future experiments, a look into relaxation behavior and potential differences between conventionally freeze-dried and microwave-assisted freeze-dried solids could be of interest. Thermal history is expected to be different. Moreover, a deeper look into potential chemical changes that may occur during MFD should be taken. For those analytical techniques focusing on structural changes like FT-IR and circular dichroism, but also methods like (peptide mapping) LC-MS, should be considered.

## 5. Conclusions

Microwave-assisted freeze-drying is an emerging technique recently introduced to the field of pharmaceutical freeze-drying of biologicals [20,24]. Despite potentially huge time savings for vial-based drying achievable by MFD [20], we were able to elucidate comparable stability profiles for different monoclonal antibody formulations over storage times of 24 weeks. Although residual moisture contents were found to be different between CFD and MFD, no adverse effect on protein stability or crystallization tendency in matrices with higher residual moisture was found. Even the occurrence of a microscopic collapse in the microwave-processed arginine phosphate mAb formulation (F3) did not lead to decreased stability, with respect to solid state- and protein-related properties. Moreover, our data indicate that with modern semiconductor solid-state microwave generators batch homogeneities of microwave batches could be approximated to those of conventional freeze-drying. However, the authors see a definite need for new machines complying with the requirements of pharmaceutical manufacturing. The new generator setup presented may open up space for engineering creativity to merge pharmaceutical needs with innovative heating techniques. 

## Figures and Tables

**Table 1:** Formulations used in this study.

Ingredient	F1	F2	F3	F4
mAb1 [g/L]	5	5	5	/
mAb2 [g/L]	/	/	/	50
Sucrose [% (*w/v*]	10	/	/	5
Trehalose [% (*w/v*)]	/	10	/	/
Arginine phosphate [% (*w/v*)]	/	/	10	/
Polysorbate 80 [% (*w/v*)]	0.02	0.02	0.02	/

Formulations F1 and F2 were formulated in 10 mM histidine buffer (pH 6.0), whereas F3 contained no additional buffer salt but was formulated and adjusted to pH 6.0. F4 was formulated in 10 mM histidine buffer (pH 5.5). mAB = monoclonal antibody.

**Table 2:** Overview of the conventional freeze-drying (CFD) processes for the respective formulations.

Freeze-Drying Process	Setpoint	Freezing			Primary Drying	Secondary Drying	
CFD cycle F1	T_Shelf_ [°C]	20	−5	−60	−20	0	20
	Ramp [K/min]	−	1	1	0.2	0.05	0.2
	Hold time [min]	5	60	2580 ^a^	2700 (1120) ^b^	−	360
	p_Chamber_ [µbar]	−	−	−	100	50	50
CFD cycle F2/3	T_Shelf_ [°C]	20	−5	−60	−25	0	20
	Ramp [K/min]	−	1	1	0.2	0.05	0.2
	Hold time [min]	5	60	3610 ^a^	5760 (1400) ^b^	-	360
	p_Chamber_ [mbar]	−	-	−	100	50	50
CFD cycle F4	T_Shelf_ [°C]	20	5	−50	−20	5	35
	Ramp [K/min]	−	1	1	1	0.15	0.3
	Hold time [min]	5	60	1006 ^a^	1868 (1058) ^b^	−	420
	p_Chamber_ [mbar]	−	−	−	100	100	100

^a^ Freezing times were held longer than usual due to logistical reasons caused by the need to split up the batch before proceeding with the drying process. ^b^ Estimated time needed for complete sublimation based on the time the last thermocouple in the respective formulation needed to reach the shelf temperature setpoint.

**Table 3:** Reconstitution times of the different formulations.

Formulation	Reconstitution Time (s)
F1	≤30
F2	≤30
F3	≤50
F4	≤120

## Supplementary Materials

The following are available online at https://www.mdpi.com/1999-4923/11/12/674/s1, Figure S1: Representative X-ray diffractograms of sucrose-based formulation F1 and F4 compared to unprocessed pure crystalline sucrose (EMPROVE® exp sucrose) from the shelf. Top graphs represent conventionally freeze-dried samples directly after FD (left) and after 24 weeks of storage at 40 °C (right). The same applies to the bottom graphs but with microwave-assisted freeze-dried samples. Figure S2: Representative X-ray diffractograms of sucrose-based formulation F1 and F4 compared to unprocessed pure crystalline sucrose (EMPROVE® exp sucrose) from the shelf. Top graphs represent conventionally freeze-dried samples directly after FD (left) and after 24 weeks of storage at 40 °C (right). The same applies to the bottom graphs but with microwave-assisted freeze-dried samples., Figure S3: Representative X-ray diffractograms of low concentration mAb formulation with arginine phosphate (F3). The diffractogram of a MFD recrystallized sample of identical composition (gray line) was used for identification of reference peaks. Top graphs represent conventionally freeze-dried samples directly after FD (left) and after 24 weeks of storage at 40 °C (right). The same applies to the bottom graphs but with microwave-assisted freeze-dried samples, Figure S4: Relative percentages of monomer and high molecular weight species (HMW) at the respective storage temperature over storage time gained by HP-SEC analysis are presented. Table S1: Residual moisture content from two similar mAb2 formulations produced with the previous microwave setup [20] compared to F4.
