# Supplementary material for: Microwave-Assisted Freeze-Drying of Monoclonal Antibodies: Product Quality Aspects and Storage Stability
Source: Pharmaceutics. 2019 Dec 12;11(12):674. doi: 10.3390/pharmaceutics11120674 (PMC6956074; doi:10.3390/pharmaceutics11120674)
Supplement: Supplementary file 1 [file pharmaceutics-11-00674-s001.pdf]

pharmaceutics

Supplementary Materials: Microwave-Assisted Freeze-Drying of Monoclonal Antibodies: Product Quality Aspects and Storage Stability

Julian Hendryk Gitter, Raimund Geidobler, Ingo Presser and Gerhard Winter

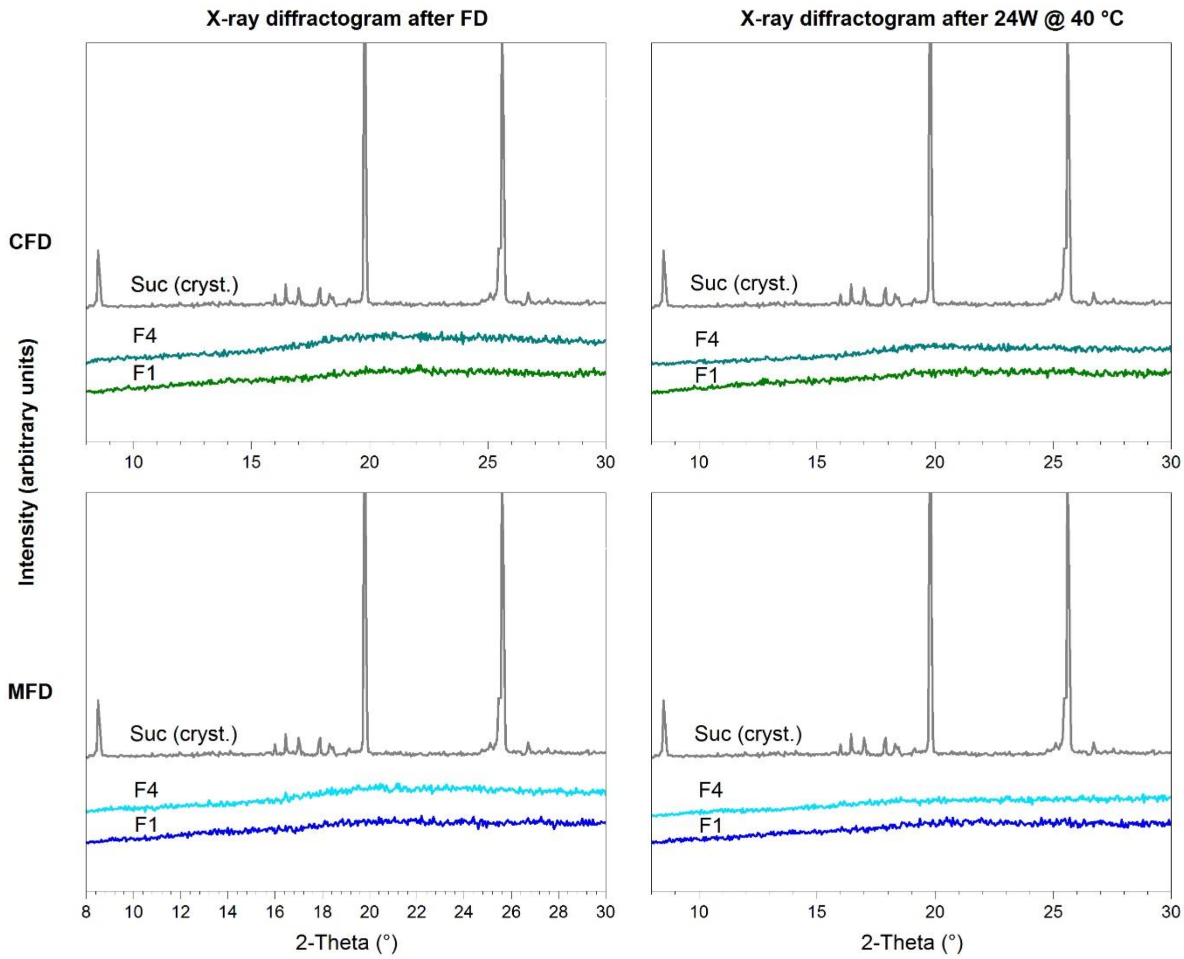

Figure S1. Representative X-ray diffractograms of sucrose-based formulation F1 and F4 compared to unprocessed pure crystalline sucrose (EMPROVE® exp sucrose) from the shelf. Top graphs represent conventionally freeze-dried samples directly after FD (left) and after 24 weeks of storage at 40°C (right). The same applies to the bottom graphs but with microwave-assisted freeze-dried samples.

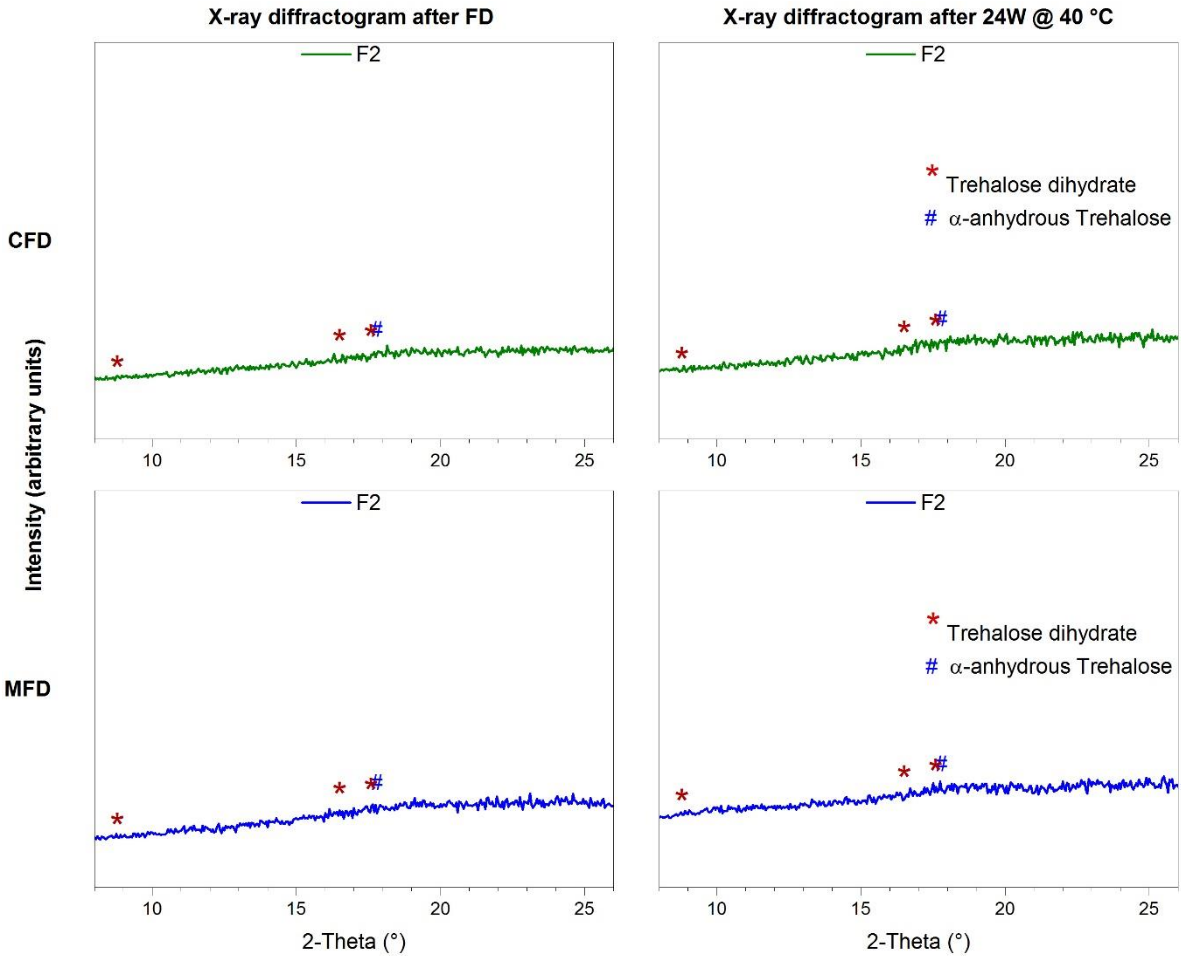

Figure S2. Representative X-ray diffractograms of trehalose-based formulation F2 marked with typical peaks related to trehalose dihydrate (asterisk) and α-anhydrous trehalose (hash) obtained from the literature [25]. Top graphs represent conventionally freeze-dried samples directly after FD (left) and after 24 weeks of storage at 40°C (right). The same applies to the bottom graphs but with microwave-assisted freeze-dried samples.

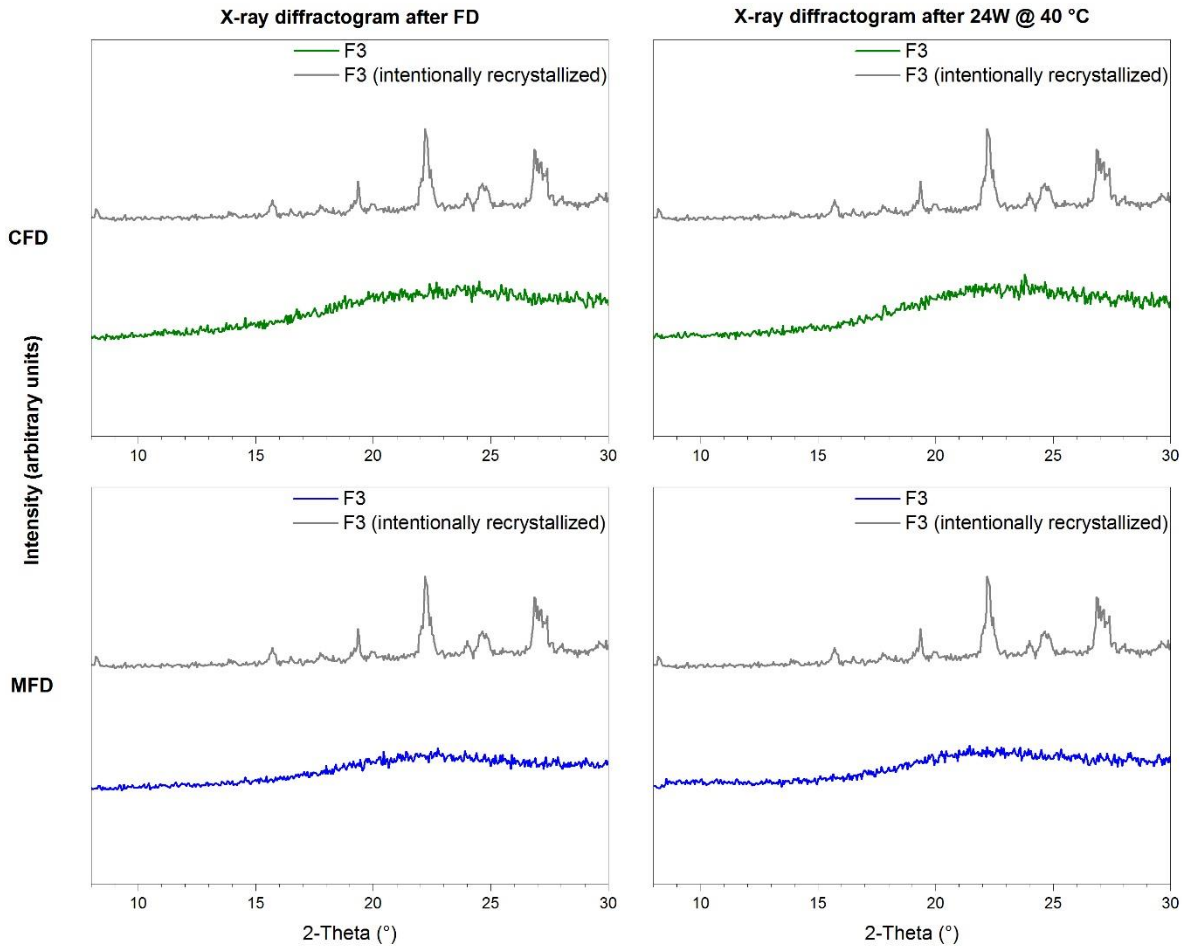

Figure S3. Representative X-ray diffractograms of low concentration mAb formulation with arginine phosphate (F3). The diffractogram of a MFD recrystallized sample of identical composition (gray line) was used for identification of reference peaks. Top graphs represent conventionally freeze-dried samples directly after FD (left) and after 24 weeks of storage at 40°C (right). The same applies to the bottom graphs but with microwave-assisted freeze-dried samples.

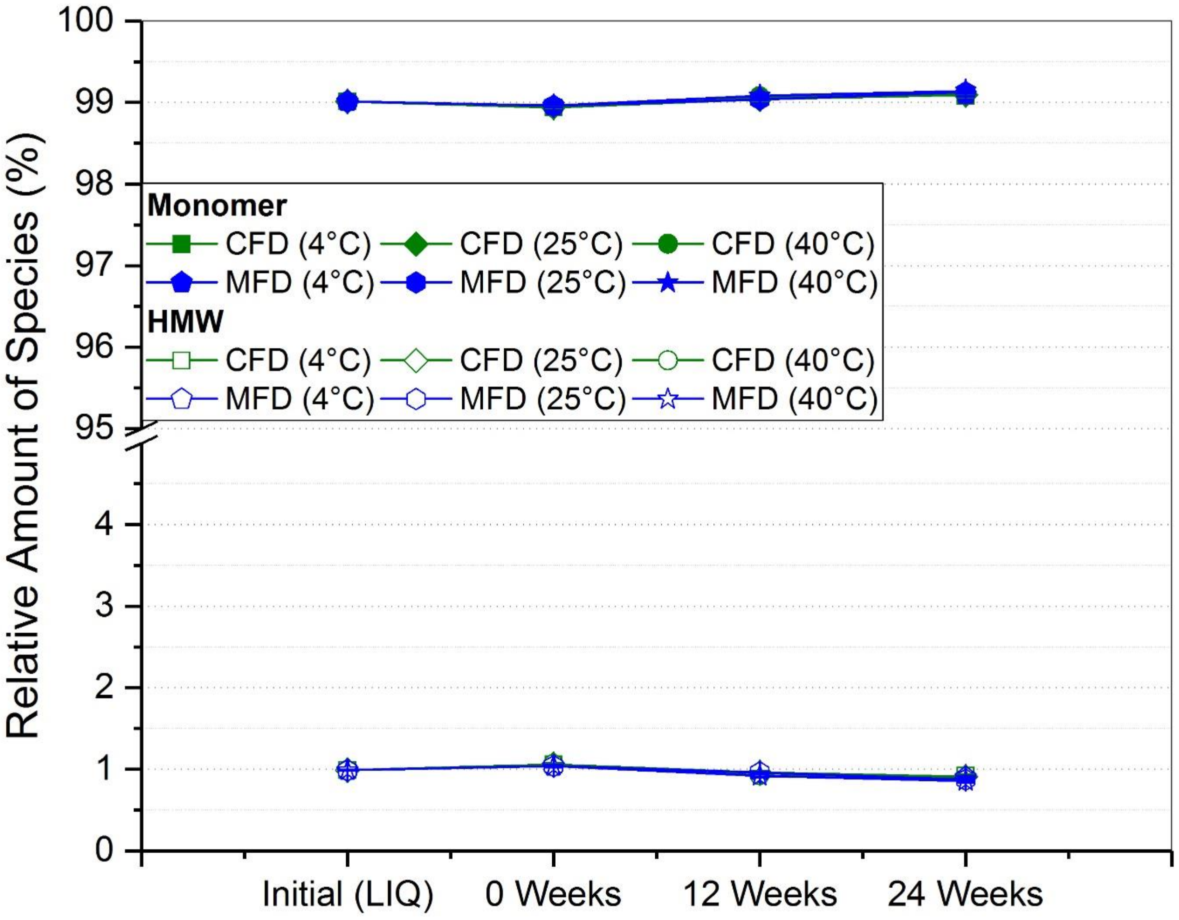

Figure S4. Relative percentages of monomer and high molecular weight species (HMW) at the respective storage temperature over storage time gained by HP-SEC analysis are presented.

Table S1. Residual moisture content from two similar mAb2 formulations produced with the previous microwave setup [20] compared to F4.

Formulation	Residual Moisture Content ± Standard Deviation (*n* = 3) [%]
**F4.1**: 40 g/L mAb2 + 6% (*w*/*v*) sucrose [a]	1.2 ± 0.7
**F4.2**: 60 g/L mAb2 + 4% (*w*/*v*) sucrose [a]	1.2 ± 1.1
**F4**	0.4 ± 0.0

[a] The formulation was buffered with a 10 mM histidine buffer pH 6.0.

Relative standard deviations of 58% and 92% were found for F4.1 and F4.2, respectively. In strong contrast to that, F4 revealed variances and derived standard deviations which were in the same order of magnitude as those for CFD samples.
